# m5C-Related lncRNAs Predict Overall Survival of Patients and Regulate the Tumor Immune Microenvironment in Lung Adenocarcinoma

**DOI:** 10.3389/fcell.2021.671821

**Published:** 2021-06-29

**Authors:** Junfan Pan, Zhidong Huang, Yiquan Xu

**Affiliations:** ^1^Shengli Clinical Medical College of Fujian Medical University, Fuzhou, China; ^2^Quanzhou First Hospital, Fujian Medical University, Quanzhou, China; ^3^Department of Thoracic Oncology, Fujian Medical University Cancer Hospital, Fujian Cancer Hospital, Fuzhou, China

**Keywords:** m5C, lncRNA, lung adenocarcinoma, prognostic signature, overall survival, tumor immune microenvironment

## Abstract

Long non-coding RNAs (lncRNAs), which are involved in the regulation of RNA methylation, can be used to evaluate tumor prognosis. lncRNAs are closely related to the prognosis of patients with lung adenocarcinoma (LUAD); thus, it is crucial to identify RNA methylation-associated lncRNAs with definitive prognostic value. We used Pearson correlation analysis to construct a 5-Methylcytosine (m5C)-related lncRNAs–mRNAs coexpression network. Univariate and multivariate Cox proportional risk analyses were then used to determine a risk model for m5C-associated lncRNAs with prognostic value. The risk model was verified using Kaplan–Meier analysis, univariate and multivariate Cox regression analysis, and receiver operating characteristic curve analysis. We used principal component analysis and gene set enrichment analysis functional annotation to analyze the risk model. We also verified the expression level of m5C-related lncRNAs *in vitro*. The association between the risk model and tumor-infiltrating immune cells was assessed using the CIBERSORT tool and the TIMER database. Based on these analyses, a total of 14 m5C-related lncRNAs with prognostic value were selected to build the risk model. Patients were divided into high- and low-risk groups according to the median risk score. The prognosis of the high-risk group was worse than that of the low-risk group, suggesting the good sensitivity and specificity of the constructed risk model. In addition, 5 types of immune cells were significantly different in the high-and low-risk groups, and 6 types of immune cells were negatively correlated with the risk score. These results suggested that the risk model based on 14 m5C-related lncRNAs with prognostic value might be a promising prognostic tool for LUAD and might facilitate the management of patients with LUAD.

## Introduction

Lung cancer is the leading cause of cancer-related deaths worldwide. Approximately, 18 million people are diagnosed with lung cancer each year, and 16 million die because of this disease (Bray et al., 2018). There are 2 main types of lung cancer: non-small cell lung cancer (NSCLC) and small cell lung cancer (SCLC). In particular, NSCLC is the main type of primary lung cancer, accounting for approximately 80–85% of cases, and it can be further classified into adenocarcinoma, squamous cell carcinoma, and large cell carcinoma (Liu et al., 2015; Osmani et al., 2018). Adenocarcinoma is the major type of NSCLC, and most patients are diagnosed when already in an advanced and inoperable metastatic stage, with a 5-y survival rate of only 4.7% (Cohen et al., 2020). Recently, targeted therapy for specific driver gene mutations and the use of immune checkpoint inhibitors have popularized individualized therapy and effectively improved the prognosis of patients with advanced metastatic disease. However, because of the high drug resistance and metastasis rate, more specific biomarkers are neededthat could lead to the development of more effective diagnostic and treatment strategies.

Methylation is one among the wide range of RNA post-transcriptional modifications, determining translation efficiency (Li Q. et al., 2017; Zhao et al., 2017). N6-methyladenosine (m6A) epitranscriptional modification is the main modification mode of RNA, and a number of high-throughput experimental methods have been developed to characterize the transcriptome range of modifications of m6A (Chen K. et al., 2019). Besides m6A, 5-methylcytosine (m5C) is another common mRNA modification (Dou et al., 2020). More specifically, m5C, in which the methyl group is attached to the fifth position of the cytosine ring, is catalyzed by RNA methyltransferase. The m5C locus has been reported to be involved in a variety of biological processes, including structural stability and metabolism of RNA, tRNA recognition, and stress response (Zhao et al., 2017). In addition, m5C modification has also been closely related to cancer progression. A recent study has shown that in human urothelial cell carcinoma of the bladder, m5C regulators bound to the 3′UTR of oncogene mRNA, stabilizing its expression, thereby promoting cancer progression (Chen X. et al., 2019). Bioinformatic studies have shown that m5C regulators could be used as a prognostic factor for lung adenocarcinoma (LUAD) (Sun L. et al., 2020), head and neck squamous cell carcinoma (Xue et al., 2020), and hepatocellular carcinoma (He et al., 2020b).

Long non-coding RNAs (lncRNAs), which are the main non-coding RNAs, are transcripts longer than 200 nt. They are known to play a key role in chromatin remodeling, transcription, and post-transcriptional regulation (Kopp and Mendell, 2018). In addition, RNA methylation of lncRNAs has been demonstrated to affect cancer progression. For instance, in hepatocellular carcinoma, the m6A “writer” METTL3 increased the stability of LINC00958, promoting the progression of cancer (Zuo et al., 2020). Similarly, the m6A “eraser” ALKBH5 increased the invasion and metastasis of tumor cells in gastric cancer by inhibiting the methylation of NEAT1 (Zhang et al., 2019). m5C modification sites are also widely distributed in non-coding RNAs. Studies revealed that progressive conventional bisulfite sequencing in HeLa cells identified new m5C candidate sites, with approximately 1780 of these sites being shown to exist in multiple types. Such non-coding RNA types include lncRNAs (Squires et al., 2012). However, there have been few studies on the regulation of m5C in lncRNAs, and further research is needed.

In recent years, a large number of studies have shown that immune cells in the tumor microenvironment (TME) play a vital role in cancer progression and the therapeutic efficacy of applied treatments (Lei et al., 2020). In the early stage of tumorigenesis, anti-tumor immune cells in the tumor microenvironment tend to target cancer cells. As the tumor progresses, tumor cells will eventually resist the cytotoxic effects of immune cells and escape immune surveillance. lncRNAs have become key regulatory elements in the immune system, playing an important role in guiding the development of a variety of immune cells and controlling the dynamic transcription program. The dynamic transcription program is a sign of immune cell activation (Sun J. et al., 2020). For example, in epithelial cells, lincRNA-Cox2 was reported to participate in the Mi-2/nucleosome remodeling and deacetylase (Mi2/NuRD) complex, facilitating the recruitment of the IL12β promoter to prevent the expression of IL12β (Atianand et al., 2017). The chromatin-related CD4^+^ Th1-specific lncRNA lnc-MAF-4 was found to be inversely proportional to the expression of the transcription factor MAF, and its downregulation elicited the differentiation of helper CD4^+^ T-cells to the Th2 phenotype (Ranzani et al., 2015). Recent studies have also emphasized that lncRNAs plays a key role in cancer immune regulation. In colorectal cancer, the antisense lncRNA SATB2-AS1 was shown to regulate SATB2 expression, inducing the expression of the CXCL9 and CXCL10 TH1-type chemokines and mediating the transport of effector T-cells (Xu et al., 2019). Based on this result, it was considered to serve as a potential target for colorectal cancer immunotherapy. In addition, a correlation between lncRNAs and immune cell infiltration has also been observed in uveal melanoma (Wang et al., 2018), pancreatic cancer (Qin et al., 2019), and cervical cancer (Guo et al., 2019). However, current research on the correlation between lncRNAs and immune cell infiltration is rare in LUAD, and further research is needed.

Although there are many bioinformatics studies focus on the RNA modifications (Chen K. et al., 2019, 2021; Liu and Chen, 2020; Liu et al., 2020; Song et al., 2020; Chen H. et al., 2021; Tang et al., 2021; Xu et al., 2021), to the best of our knowledge, this is the first in-depth analysis of the role of m5C regulators in LUAD. In this study, we used the expression data of m5C-related lncRNAs inLUAD from the Cancer Genome Atlas(TCGA)dataset, screened out 14 m5C-related lncRNAs with prognostic value, constructed an m5C-related lncRNAs prognostic signature (m5C-LPS), and further analyzed the relationship between m5C-LPS and immune cell infiltration subtypes. We aimed to explore the different gene characteristics, prognostic value, and impact on the tumor immune microenvironment (TIM) of the RNA methylation of m5C-related lncRNAs in LUAD, so as to provide guidance for the treatment of LUAD.

## Materials and Methods

### Data Acquisition

Transcriptome analysis of raw data and corresponding clinical information of the LUAD cohort were downloaded from TCGA data portal^1^. A total of 551 patients with LUAD with lncRNA expression profiles, including 497 LUAD tissues and 54 normal lung tissues, of which 486 cases contained follow-up time and complete clinical case characteristics. Table 1 lists the detailed clinical characteristics of the 486 patients with LUAD. lncRNA annotation file of Genome Reference Consortium Human Build 38 (GRCh38) was acquired from the GENCODE website for annotation of the lncRNAs in the TCGA dataset. Based on recognizing the Ensemble IDs of the genes, 14,142 lncRNAs were identified in the TCGA dataset.

**TABLE 1:** The clinical characteristics of lung adenocarcinoma patients in the TCGA database.

Variables	No. of Patients	Percentage (%)
Age (years)		
≤65	227	46.7
>65	240	49.4
Unknown	19	3.9
Gender		
Female	264	54.3
Male	222	45.7
Pathological stage		
I	262	53.9
II	112	23.0
III	79	16.3
IV	25	5.1
Unknown	8	1.7
T stage		
T1	163	33.5
T2	260	53.5
T3	41	8.5
T4	19	3.9
Unknown	3	0.6
N stage		
N0	312	64.2
N1	90	18.5
N2	70	14.4
N3	2	0.4
Unknown	12	2.5
M stage		
M0	333	68.5
M1	24	4.9
Unknown	129	26.6

### Identification of Differentially Expressed Genes in TCGA Database

A total of 13 m5C regulators were obtained from the published literature, comprising *NSUN2, NSUN3, NSUN4, NSUN5, NSUN6, NSUN7, ALYREF, DNMT1, DNMT3A, DNMT3B, TET2, TRDMT1*, and *YBX1*. Extract of the expression matrix and clinical data of m5C regulators of 551 patients with LUAD were obtained from TCGA database. Then, the limma software package in R version (4.0.2) was used to identify the differentially expressed m5C regulators between LUAD and normal lung tissues. A *p* value < 0.05 and | log2 (folding change)| > 1 were considered significantly different. Subsequently, heatmaps were used to show the differential expression of m5C regulators between LUAD and normal lung tissues.

### Construction of Protein–Protein Interaction (PPI) Network

We analyzed the differentially expressed 13 m5C regulators using the Search Tool for Interaction Genes (STRING) database^2^ to construct a PPI network (Li et al., 2020). As a threshold of genes at the center of the PPI network was set a minimum gene interaction score < 0.7.

### m5C-Related lncRNAs

The “limma R” package was used to detect m5C-related lncRNAs. The m5C-related lncRNAs were identified by the correlation analysis between the m5C genes and lncRNA expression levels in the LUAD samples. The correlation coefficient > 0.3 and *p* < 0.001 as criteria, and 1094 m5C-related lncRNAs were identified.

### Constructing the Prognostic Risk Model of m5C-Related lncRNAs

In order to identify m5C-related lncRNAs with prognostic value, We conducted a univariate Cox analysis based on the standard of *p* < 0.01. Subsequently, multivariable Cox analysis was used to establish a risk score. The risk score of each patient was calculated according to the following formula:

Risk score = coef (lncRNAn) × expr (lncRNAn), where coef (lncRNAn) and expr (lncRNAn) represent the survival correlation regression coefficient and expression value, respectively, of each m5C-related lncRNA.

It should be noted that coef (lncRNAn) was defined as the correlation between lncRNAs and survival, whereas expr (lncRNAn) was defined as the expression of lncRNAs.

### Evaluation of the Risk Model of 14m5C-Related lncRNAs as Independent Prognostic Factor in LUAD

According to the median value of the prognostic risk score, patients with LUAD were divided into high- and low-risk groups. The Kaplan–Meier survival curve was used to compare the overall survival (OS) of patients in the two groups. Principal component analysis (PCA) was conducted for effective dimensionality reduction, pattern recognition, and exploratory visualization analysis of the whole genome, m5C-related coding genes, and m5C-related lncRNAs expression profiles. Univariate and multivariate Cox regression analyses were used to assess whether the risk score was independent of other clinical variables. The receiver operating characteristic (ROC) curve was used to evaluate the diagnostic and prognostic value of clinicopathological features. A *p* value < 0.05 was considered statistically significant.

### Gene Set Enrichment Analysis (GSEA)

Gene set enrichment analysis was performed in the LUAD cohort to gain insights into the biological pathways of the high- and low-risk subgroups defined by the expression characteristics of the 14 m5C-related lncRNAs. Gene sets with false discovery rate (FDR) < 0.25 and normalized *p* value < 0.05 were considered significant.

### Cell Lines and Reagents

Human normal lung epithelial cell line BEAS-2B, human LUAD cell lines PC-9, H1299 were purchased from the Cell Room of School of Medicine, Central South University. All cells were cultured in RPMI 1640 medium (Gibico, United States) supplemented with 10% fetal bovine serum (10% FBS), and the cells were cultured in a humidified atmosphere at 37°C and 5% CO_2_.

### Total RNA Extraction and Real-Time Quantitative PCR

In order to evaluate the expression level of m5C-related lncRNAs, we used RNA trizol reagent (Invitrogen, Carlsbad, CA, United States) to isolate total cellular RNA. Reverse transcription was done using PrimeScript^TM^ RT reagent Kit (Takara, Japan). Real-time fluorescent quantitative PCR was performed by using GoTaq^®^ qPCR Master Mix kit (Promega, United States). The expression level of related lncRNAs was calculated using 2^–ΔΔ*CT*^, and the related GAPDH mRNA expression was used as an endogenous control. The primer sequences involved in this study are shown in Supplementary Table 1. Each PCR reaction was performed in triplicate.

### Prediction of m5C Sites on 14 lncRNAs

RNAm5Cfinder^3^ (Li et al., 2018), iRNAm5C-PseDNC^4^ (Qiu et al., 2017), iRNA-m5C^5^ (Lv et al., 2020) databases were used to further verify the 14 prognostic-related lncRNAs were m5C modified lncRNAs by predicting m5C modification sites on lncRNAs.

### Analysis of Immune Cell Characteristics

CIBERSORT is an analytical tool with a gene expression signature matrix of 547 marker genes used for the quantitative infiltration of immune cell components. LM22, which defines the 22 immune cell subtypes of the annotation of the gene signature matrix, was downloaded from the CIBERSORT website^6^. We used 100 permutations of the default signature matrix to calculate the CIBERSORT *p* value and root-mean-square error for each sample file to improve the accuracy of the deconvolution algorithm. Then, we used the CIBERSORT value of *p* < 0.05 and data of LUAD tissues were screened and selected for subsequent analysis. The CIBERSORT algorithm was used to analyze the immune cell composition of LUAD samples in TCGA cohort.

### The TIMER Database

In order to assess the risk score and the level of correlation of the infiltration of immune cells, we used the TIMER database^7^. This database contains 10897 TCGA samples of 32 types of cancer. Data were collected on 6 types of immune infiltrates, including B-cells, CD4^+^ T-cells, CD8^+^ T-cells, neutrophils, macrophages, and dendritic cells. In addition, it provides three main analysis modules: Immune, Exploration, and Estimation. The Immune module includes clinical outcomes, somatic mutation, and somatic copy number change, enabling users to comprehensively analyze the relationship between immune cell infiltration and multiple factors (Li T. et al., 2017).

### Statistical Analysis

One-way analysis of variance was used to compare the expression levels of 13 m5C regulators in 497 LUAD tissues and 54 normal lung tissues. A coexpression network of 14 prognostic m5C-related lncRNAs-mRNAs was established and visualized using Cytoscape. Gene Expression Profiling Interactive Analysis (GEPIA) was used to evaluate the correlation between m5C-related lncRNAs and m5C regulators. The ggpubrpackage in Rwas used to analyze the correlation between the expression of 14 m5C-related lncRNAs and clinicopathological factors. PCA was used to effectively reduce dimensionality, pattern recognition, and exploratory visualization of high-dimensional data for the whole genome, m5C-related coding genes, and 14 m5C-related lncRNAs expression profiles. GSEA was performed for functional annotation. The Kaplan–Meier method was used to compare the OS time of each group. Univariate and multivariate Cox proportional hazards models were used to determine significant prognostic factors. ROC curve was used to evaluate the predictive efficiency of the prognostic risk scoring model. The RT- qPCR results were analyzed using One-way ANOVA. Statistical analysis was performed using R software (version 4.0.2). A *p* < 0.05 was considered statistically different.

## Results

### Differentially Expressed m5C Regulators Between LUAD and Normal Lung Tissues

**FIGURE 1:**
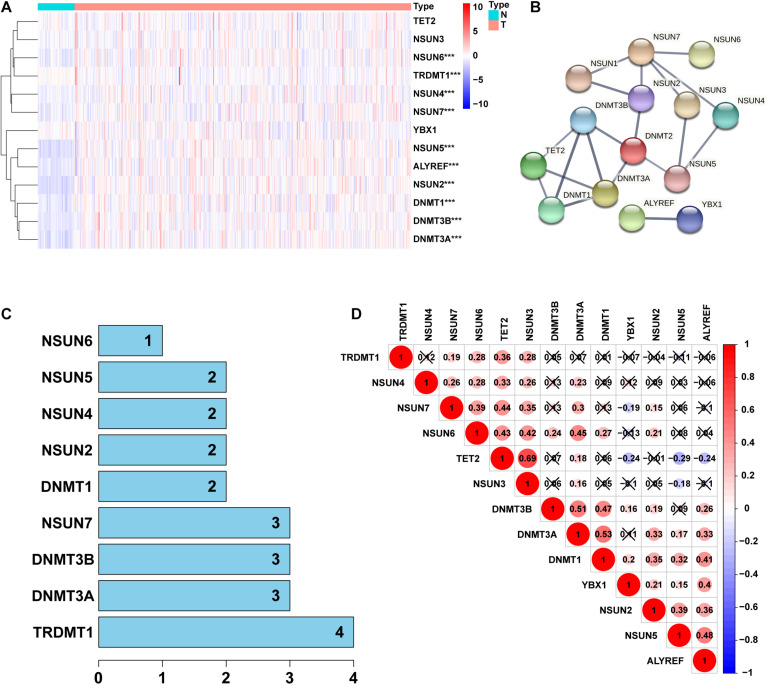
Identification of differentially expressed genes between 5-Methylcytosine (m5C) regulators in lung adenocarcinoma (LUAD) and normal groups. **(A)** Heatmap visually showing the differences in the expression of m5C regulators between the 2 groups. **(B,C)** Protein–protein interaction (PPI) network showing the interaction between differentially expressed genes among m5C regulators. **(D)** Pearson correlation analysis of the m5C regulators. N, normal samples; T, tumor samples; ***p < 0.001.

To identify the differential expressed genes and related functions of m5C regulatory factors in LUAD, we used TCGA database to analyze 497 and 54 cases of LUAD and normal lung tissues, respectively. Our results showed that the expression of m5C regulators was significantly different between LUAD and adjacent normal tissues (Figure 1A). We found that among all genes tested, the expression of *NSUN2, NSUN5, DNMT3B, DNMT3A, ALYREF, DNMT1, NSUN6, NSUN4*, and *NSUN7* were significantly higher in LUAD than normal tissues (*p* < 0.001). We also observed that the expression of *TRDMT1* in cancer tissues was significantly lower than that in the normal group (*p* < 0.001), whereas no significant differences were noted between the groups in the expression of *NSUN3, YBX1*, and *TET2* (Supplementary Table 2). Next, we tried to clarify the relationship between m5C regulators. We used the STRING database to construct a PPI network of 14 m5C-related lncRNAs (Figure 1B), and the number of nodes were showned in Figure 1C. *TRDMT1* was the core gene of the network and was found to interact with 4 other genes. However, when we performed the correlation analysis, *TRDMT1* did not show a strong correlation with other genes. In addition, we observed that *NSUN4, NSUN7, NSUN6, DNMT3B, DNMT3A*, and *DNMT1* had weak to moderate correlation with other genes, whereas *NSUN3* and *TET2* had the strongest correlation (Figure 1D). The above results indicated that there was a certain interaction between m5C regulators in LUAD.

### Identification of m5C-Related lncRNAs With Important Prognostic Value

**FIGURE 2:**
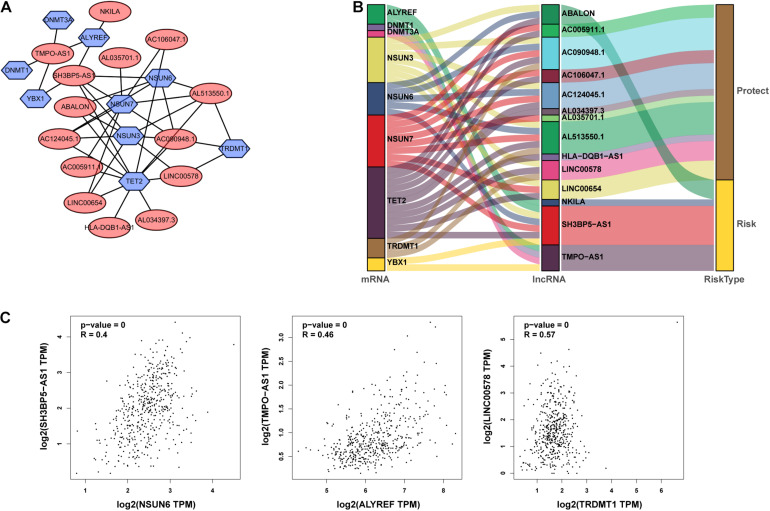
Identification of m5C-associated lncRNAs with significant prognostic value in LUAD. **(A,B)** Coexpression network of m5C-related lncRNAs–mRNAs. **(C)** Expression intensity of related genes in the coexpression network analyzed using the Gene Expression Profiling Interactive Analysis (GEPIA) online database.

From TCGA database, we identified 1094 m5C-related lncRNAs combined with LUAD survival data and performed univariate Cox regression analysis to determine the m5C-related lncRNAs with important prognostic value. Accordingly, our results revealed53 m5C-related lncRNAs associated with cancer risk (*p* < 0.05) (Supplementary Table 3). Among them, 46 m5C-related lncRNAs were demonstrated to be protective factor (HR < 1), whereas7of them were high-risk factors (HR > 1). Furthermore, we used multivariate Cox regression analysis;of the 53 m5C-related lncRNAs, 14 m5C-related lncRNAs were revealed to have prognostic value (Table 2). As shown in Figures 2A,B, we constructed a coexpression network for the visualization of 14 m5C-related lncRNAs-mRNAs in LUAD. We detected that the highest number of lncRNAs was coexpressed with *TET2* (*n* = 11), followed by *NSUN7* (*n* = 8) and *NSUN3* (*n* = 7). The highest number of mRNAs was shown to be coexpressed with SH3BP5-AS1 (*n* = 6), with the coexpressed lncRNAs being the main protective factors. Then, we used the GEPIA online database to analyze the expression intensity of related genes in the co-expression network (*R* ≥ 0.35) (Figure 2C and Supplementary Figure 1). We found that there was a weak correlation between m5C-related lncRNAs and m5C genes, among which the strongest correlation was found between *TRDMT1* and *LINC00578* (*R* = 0.57, *p* < 0.001), followed by *ALYREF* and *TMPO-AS1* (*R* = 0.46, *p* < 0.001), with the expression of m5C-related mRNAs being positively correlated with the expression of lncRNAs.

**TABLE 2:** The 14 m5C-Related Prognostic lncRNAs.

ID	Coef	HR	HR. 95L	HR. 95H	*P* value
AC124045.1	−0.487330859	0.614263765	0.404713916	0.932312821	0.022068291
NKILA	0.103614091	1.109172333	1.048491764	1.173364738	0.000306694
AC090948.1	−0.504854345	0.603593486	0.35583685	1.02385432	0.061133855
AL035701.1	−0.108845737	0.89686876	0.78069115	1.030335201	0.124106608
LINC00578	−0.156948523	0.854748057	0.763901987	0.95639788	0.006189461
AC106047.1	−0.304007158	0.737855585	0.542076261	1.004343674	0.05330851
ABALON	0.26512182	1.303589769	0.917226317	1.852701188	0.139348063
HLA-DQB1-AS1	−0.047113201	0.9539794	0.907522841	1.002814094	0.064365554
AC005911.1	−0.339018709	0.71246912	0.482910828	1.051151097	0.087533584
AL513550.1	−0.377572971	0.685523176	0.499373156	0.941063851	0.019504797
AL034397.3	−0.19339043	0.824160135	0.66003064	1.02910363	0.087863805
TMPO-AS1	0.299437423	1.349099622	1.105221645	1.646791662	0.003246416
SH3BP5-AS1	0.239153168	1.270173071	1.078451102	1.495978471	0.004174978
LINC00654	−0.32999152	0.71892983	0.564726421	0.915239807	0.007384148

**FIGURE 3:**
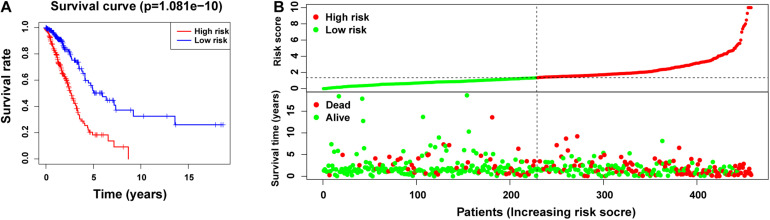
Prognostic value of risk models based on m5C-related lncRNAs. **(A)** Kaplan–Meier curve showing that the overall survival of the high-risk group was lower than that of the low-risk group (*p* < 0.001). **(B)** Risk score distribution (based on m5C-related lncRNAs prognostic signature (m5C-LPS) and survival status in patients with LUAD.

Subsequently, we divided 1344 patients with LUAD into high-and low-risk groups according to the obtained risk score formula and median risk values. Kaplan–Meier survival analysis showed that the OS of the high-risk group was poorer than that of the low-risk group (*p* < 0.001) (Figure 3A), indicating that the risk score had a prognostic value. We then drew risk curves and scatter plots to illustrate the relationship between the risk score of patients with LUAD and the corresponding survival status (Figure 3B), which suggested that the higher the risk score, the higher the mortality rate. Hence, we identified m5C-related lncRNAs with important prognostic value and established the prognostic value of m5C-LPS based on the 14 m5C-related lncRNAs.

### Correlation Between Differential Expression of m5C-Related lncRNAs and Clinicopathological Variables

**FIGURE 4:**
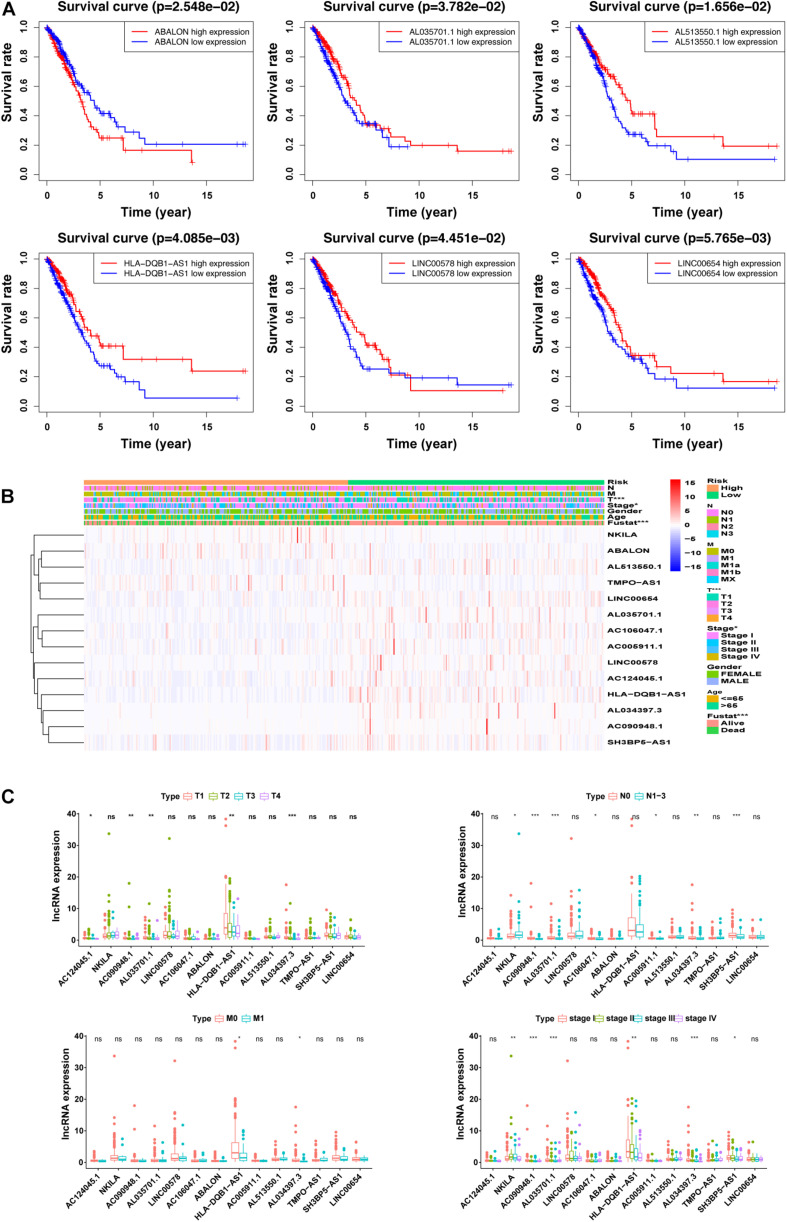
Correlation between the expression of the 14 m5C-related lncRNAs and clinicopathological factors. **(A)** overall survival (OS) analysis of the 14 m5C-related lncRNAs in the GEPIA database between high- and low-expression groups. **(B)** Heatmap showing clinicopathological features and differences in gene expression in the high- and low-risk group. **(C)** Differences in the expression of the 14 m5C-related lncRNAs in T, N, M, and S stage groups. *p < 0.5, **p < 0.01, and ***p < 0.001. ns, no sense.

As shown in Figure 4A and Supplementary Figure 2, we performed univariate analysis on the 14m5C-related lncRNAs and divided the patients into high- and low-expression groups according to the expression of single genes. We also observed significant differences in the OS of patients. Among them, the OS of patients in the high-expression group of *ABALON, AL034397.3, NKILA*, and *TMPO-AS1* was demonstrated to be lower than that in the low-expression group (*p* < 0.05). In contrast, the OS of patients in the high-expression group of *AC005911.1,AC090948.1, AC106047.1, AC124045.1, AL513550.1, HLA-DQB1-AS1, LINC00654, AL035701.1, LINC00578, and SH3BP5-AS1* was higher than that in the low-expression group (*p* < 0.05). According to the heatmap, the pathological stage (*p* < 0.05), T stage (*p* < 0.001), and survival status (*p* < 0.001) were significantly different between the high-and low-risk groups of m5C-LPS. However, we did not find any significant differences in gender, age, N stage and M stage (Figure 4B). Next, we performed an in-depth analysis of the correlation between m5C-related lncRNAs and clinicopathological features and found that in T stage, 5 lncRNAs exhibited significant differences across groups. Likewise, 7 lncRNAs were found to show significant differences among different groups in the N stage. Regarding M and S stages, 2 and 6 lncRNAs, respectively, were identified with significant differences among different groups. Interestingly, we detected that the expression of *AL034397.3* varied across groups in all (T, N, M, and S) stages, suggesting that this might be the core prognostic gene (Figure 4C).

### Verification of the Prognostic Model Constructed With the m5C-Related lncRNAs and Construction of the Nomogram

**FIGURE 5:**
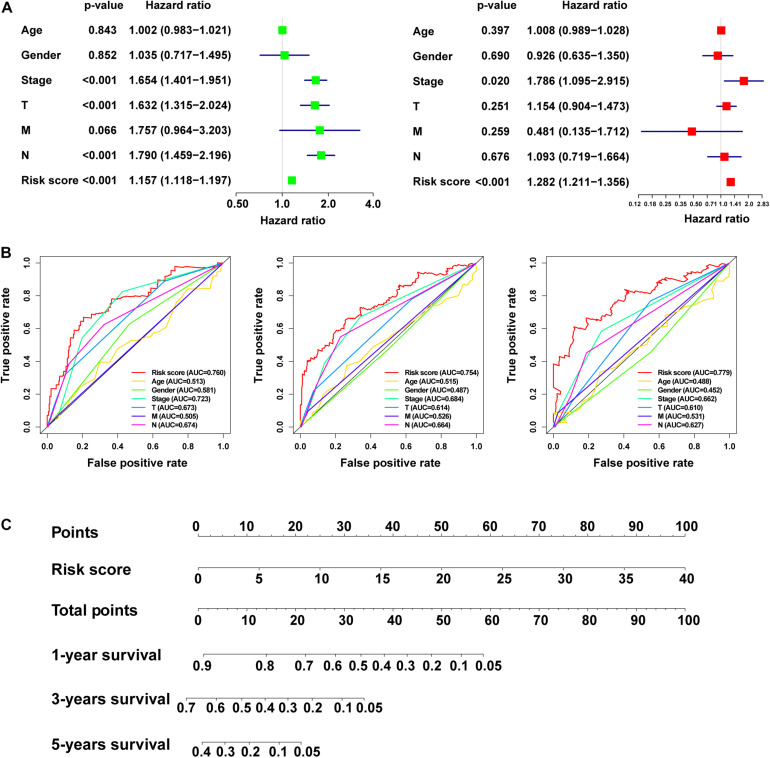
Verificationof the risk model and construction of the nomogram. **(A)** Univariate and multivariate Cox regression analysis of the prognostic value of risk scores and clinical features. **(B)** Determination of the area under the ROC curve (AUC) of the risk score and clinical characteristics based on the ROC curve. **(C)** Construction of the nomogram based on the risk score.

We used univariate and multivariate Cox regression analysis to identify whether m5C-LPS could be used as an independent prognostic factor. In the univariate Cox regression analysis, we obtained an HR = 1.157 and 95% CI: 1.118–1.197 of the risk score (*p* < 0.001), whereas in the multivariate Cox regression analysis, an HR = 1.282 and 95% CI: 1.211–1.356 of the risk score (*p* < 0.001) was achieved, indicating that the risk score as an important prognostic factor could be independent of age, gender, pathological stage, and TNM stage (Figure 5A). In order to evaluate its specificity and sensitivity for predicting the prognosis of patients with LUAD, we evaluated the area under the ROC curve (AUC) value of the risk score. We found that the AUC values of the risk score at 1, 3, and 5 years were 0.760, 0.754, and 0.779, respectively; these were demonstrated to be higher than those of other clinicopathological factors (Figure 5B). The above results indicated that m5C-LPS was a significant independent prognostic factor for patients with LUAD. At the same time, we used the risk score to construct the nomogram, which can be used as a quantitative tool to predict the prognosis of patients in clinical practice (Figure 5C).

### Differences in the m5C Status of Low- and High-Risk Groups

**FIGURE 6:**
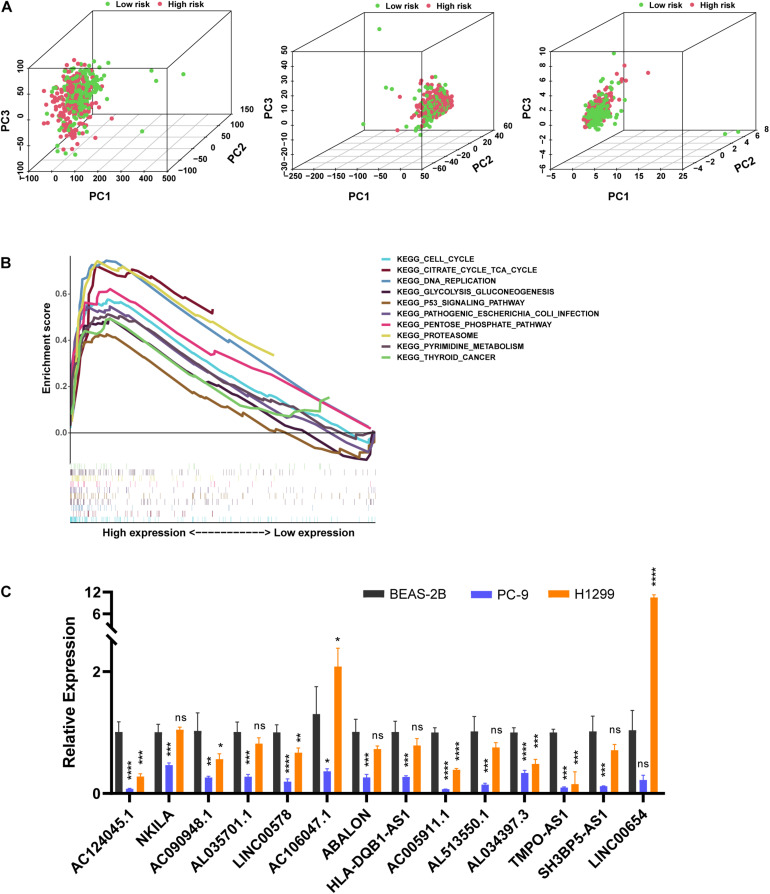
The m5C status was different between the high- and low-risk groups and the expression of m5C-related lncRNAs differed *in vitro*. **(A)** Principal component analysis (PCA) was performed for the low- and high-risk groups based on the whole genome and m5C-related coding genes, and a risk model was constructed using 14 m5C-related lncRNAs. **(B)** Gene set enrichment analysis of m5C-related lncRNAs. **(C)** Expression level of m5C-related lncRNAs in BEAS-2B cells and LUAD cells. *p < 0.5, **p < 0.01, ***p < 0.001, and **** *p* < 0.0001. ns, no sense.

We used PCA to detect the different distribution patterns of m5C on the genome-wide expression profile, m5C RNA modification-related gene expression profile, and m5C-LPS related lncRNAs expression profile between the high- and low-risk groups (Figure 6A). Based on m5C-LPS, patients were divided into low-and high-risk groups, which were distributed in different directions in a more obvious manner than when the other 2 methods were used. Hence, m5C-LPS could divide patients with LUAD into 2groups, indicating the sensitivity and specificity of the predictive model. To identify the abnormally activated signal pathways of m5C-related lncRNAs, we conducted a GSEA. the results of our analysis showed that the high expression of m5C-related lncRNAs was related to cell cycle signaling pathways, DNA replication, and *P53* signaling pathways (Figure 6B).

### Verification of Expression Level *in vitro* and Predicting m5C Modification Sites on 14 Prognostic lncRNAs

In order to verify the expression level of prognostic m5C-related lncRNAs in LUAD cells, we used RT-qPCR analysis to detect BEAS-2B and LUAD cells (Figure 6C). Among them, 9 lncRNAs (AC090948.1, AC124045.1, AL513550.1, AC005911.1, AL034397.3, LINC00578, AL035701.1, HLA-DQB1-AS1, and SH3BP5-AS1) were downregulated in both LUAD cells, combined with Figure 4A, Supplementary Figure 2, and Table 2, their high expression were associated with better survival, HR < 1, considering that they maight play a role as tumor suppressor genes. However, LINC00654, AC106047.1, and NKILA were downregulated in PC-9 cells and upregulated in H1299 cells. ABALON and TMPO-AS1 were downregulated in both LUAD cells, However, their low expression were associated with better survival, HR > 1, so the specific mechanism needed to be further studied. We further studied the m5C modification sites on prognostic m5C-related lncRNAs using online databases, and it was found that there were m5C modification sites on 14 lncRNAs (Supplementary Table 4). However, there were some differences in the results of the three databases, which might be caused by different calculation and prediction methods.

### Correlation Between m5C-LPS and Tumor Immune Microenvironment of LUAD

**FIGURE 7:**
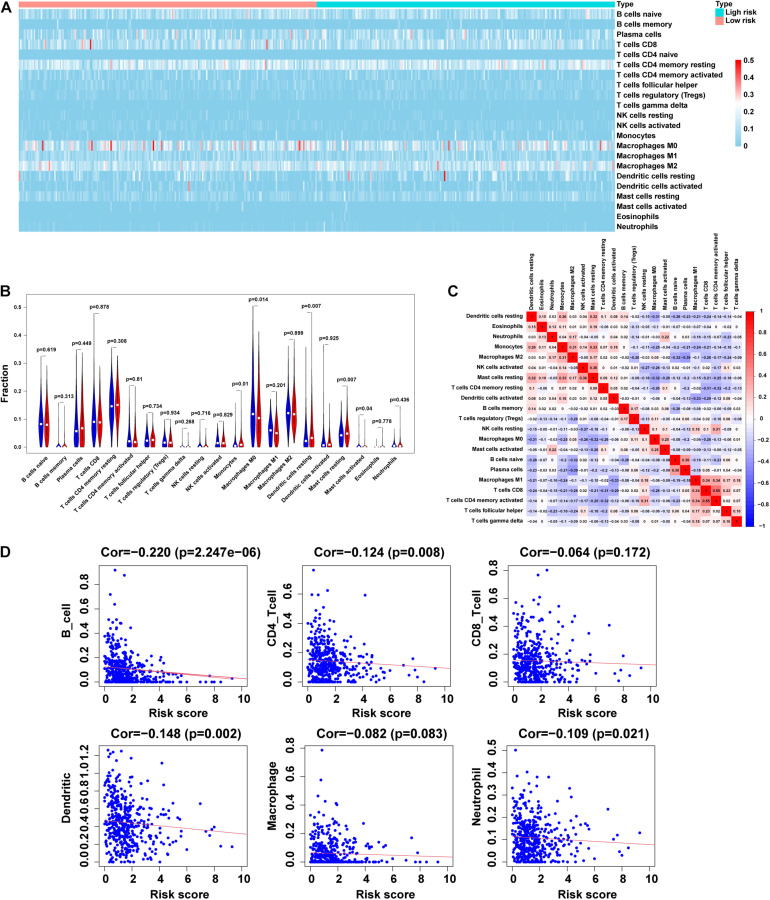
Correlation between tumor-infiltrating immune cells and risk model. **(A,B)** Heatmap and violin plot of 21 tumor-infiltrating immune cell types in low- and high-risk groups. **(C)** Spearman correlation analysis of 21 tumor-infiltrating immune cells. **(D)** Correlation of risk score with 6 tumor-infiltrating immune cell subtypes.

In this study, we analyzed the importance of TIM based on the risk characteristics of m5C-LPS. By using CIBERSORT to screen for values of *p* < 0.05, CD4 naive T-cells were not expressed in the high and low risk groups and were excluded (Figure 7A). Therefore, we analyzed the correlation between 21 tumor-infiltrating immune cells and m5C-LPS. We accordingly found that among them, monocytes, dendritic cell-resting, and mast cell-resting exhibited a higher expression in the high-risk group than in the low-risk group (*p* < 0.05). In contrast, the levels of activated M0 macrophages and mast cells in the high-risk group were lower than those in the low-risk group (*p* < 0.05) (Figure 7B). In addition, we further analyzed the correlation between 21 types of tumor-infiltrating cells. We identified that activated CD4 memory T-cells had the strongest correlation with CD8 T-cells (*r* = 0.55), followed by plasma cells and M2 macrophages (*r* =−0.39) (Figure 7C). Finally, we studied the relationship between the m5C-LPS risk score and tumor-infiltrating immune cells. Our results showed that the risk score was negatively correlated with 6 tumor-infiltrating immune cell subtypes (B-cells, CD4^+^T-cells, CD8^+^T-cells, macrophages, neutrophils, and dendritic cells; *p* < 0.05) (Figure 7D). These findings indicated that the risk characteristics of m5C-LPS could distinguish different characteristics of tumor immune cells in LUAD.

## Discussion

Although active multimodal therapy (surgery, chemotherapy, radiotherapy, targeted therapy, and immunotherapy) has greatly improved the survival of patients with LUAD, the outcome of treatment remains unsatisfactory. Patients with similar clinical risk factors have very different prognoses and tumor responses to treatment. Thus, finding effective therapeutic targets is of great value for the diagnosis and treatment of LUAD. More than 150 RNA post-transcriptional modifications have been discovered, with m6A, m5C, and m1A being the most common ones. They have been reported to play an important role in regulating gene expression and disease progression. However, abnormal RNA modification has been shown to cause a series of diseases, including cancer (Esteve-Puig et al., 2020). In this study, we used TCGA database and STRING to analyze the differences in the expression of 13 m5C regulators in LUAD and protein interactions, respectively. To the best of our knowledge, this is the first in-depth analysis of the role of m5C regulators in LUAD.

Long non-coding RNAs are widely expressed in human cells, playing a key role in various biological events, such as genome expression and cell differentiation (Wahlestedt, 2013; Luo et al., 2015; Ballantyne et al., 2016). Increasing evidence have indicated that the abnormal expression of lncRNAs might be related to the occurrence and development of a variety of cancers (Dong et al., 2018; Zhuang et al., 2019; Zhang et al., 2020). For example, the m6A “writer” METTL14 was reported to inhibit the proliferation and metastasis of colorectal cancer through the downregulation of the oncogenic XIST lncRNA (Yang et al., 2020). The lncRNA activating regulator of DKK1 (LNCAROD) was shown to be stabilized by m6A methylation in HNSCC cells and suggested to be used as a scaffold to promote the YBX1–HSPA1A PPI and stability of the YBX1 protein, resulting in the cell proliferation and migration of head and neck squamous cell cancer (Ban et al., 2020). At present, few studies exist on m5C-related lncRNAs. Studies have performed quantitative mapping of the m5C sites in Arabidopsis thaliana on a transcriptome range, and found more than 1000 m5C sites in mRNA, long non-coding RNA and other non-coding RNAs (David et al., 2017). He et al. (2020a) found that compared with adjacent non-tumor tissues, lncRNA m5C methylation occurs more frequently in Hepatocellular Carcinoma(HCC), and more methylated genes are up-regulated. KEGG pathway enrichment analysis show that hypermethylated genes are closely related to cancer pathways. It is speculated that m5C may be involved in the occurrence and development of HCC. Recent studies have found that H19 lncRNA modified by m5A “writer” NSUN2 might promote the occurrence and development of hepatocellular carcinoma by recruiting the G3BP1 oncoprote in and MYC protein (Sun Z. et al., 2020). However, there have been no reports on the role of m5C-related lncRNA in LUAD.

In this study, we analyzed the correlation between the expression of 1094 m5C-related lncRNAs and the prognosis of LUAD. In addition, 14 m5C-related lncRNAs with prognostic value were identified. Using multivariate Cox and risk scoring methods, we developed a m5C-LPS that divided patients with LUAD into high- and low-risk groups with significantly different OS. In addition, univariate and multivariate Cox analysis and AUC values of ROC curves confirmed that the risk score based on the expression of the 14 m5C-related lncRNAs could predict patient prognosis independently of traditional clinical risk factors and molecular characteristics. The currently constructed LUAD risk scoring model was mainly based on whole-genome sequencing, including lncRNA, miRNA, and mRNA. When exploring the potential of lncRNAs as novel tumor biomarkers, previous studies have focused on single molecules. However, a lncRNA is not enough to serve as a new type of malignant tumor biomarker. As lncRNAs have been reported to be released in various body fluids, including serum, saliva, and urine, it could be assumed that lncRNAs exist stably in human serum/plasma. A number of studies have reported on the differences in the expression of lncRNAs in serum/plasma as a new type of biomarker in assessing patients. However, this is the first study to report a LUAD risk score model based on 14 m5C-related lncRNAs with prognostic value. In this study, 14 m5C-related lncRNAs were selected, 4of which have been previously studied in NSCLC (Lu et al., 2017; Zhou et al., 2019; Jin et al., 2020). Bioinformatic analysis showed that HLA-DQB1-AS1 and AL034397.3 were immune-related lncRNAs in LUAD, and the constructed risk model had prognostic value.

Furthermore, lncRNAs are known to play a vital role in the TIM. The value of immune-related lncRNAs has been shown in many cancers, such as hepatocellular carcinoma (HCC), diffuse large B-cell lymphoma, and breast cancer. Currently, a number of studies have focused on 7 immune-related lncRNAs (AC022784-1, NKILA, AC026355-1, AC068338-3, LINC01843, SYNPR-AS1, and AC123595-1) to construct a LUAD risk score model, which could effectively predict clinical prognosis. In this study, we further analyzed the correlation between m5C-LPS and the distribution of tumor-infiltrating immune cells. We found that the risk score was negatively correlated with the degree of these6 immune cell infiltrates. Our results show that the risk characteristics of m5C-LPS could distinguish different characteristics of tumor-infiltrating immune cells in LUAD. As such, this study is the first to analyze the role of m5C-related lncRNAs in LUAD and their correlation with TIM.

However, we also recognize some limitations of this study. For example, the dataset used for the initial analysis was relatively insufficient. We only downloaded the data from TCGA and failed to retrieve other lncRNA expression levels supporting LUAD, patient clinicopathological characteristics, survival, and follow-up data. In addition, the nomogram we constructed only included the risk score, because the patients of stage I and II were far more than those of stage III and IV in the TCGA, so we excluded the stage and constructed a nomogram using risk score. We only validated the prognostic m5C-related lncRNAs at the cytological level. The constructed risk scoring model needs to be validated by tissue level and *in vivo* experiments to make the prediction results more reliable. Additionally, there was a lake of experiments such as MeRIP-seq, RNA-BisSeq, Aza-IP, miCLIP and other experiments to further confirm m5C modification sites on 14 lncRNAs. We will incorporate this work into future research.

## Conclusion

The m5C-LPS we constructed was demonstrated to have an independent prognostic value and high reliability and might provide some clues for further research on the mechanism of the m5C post-transcriptional modification of lncRNAs, and at the same time bestow new resources for a better understanding of the mechanism of immune cell-specific genes involved in the regulation of cancer.

## Data Availability Statement

The datasets presented in this study can be found in online repositories. The names of the repository/repositories and accession number(s) can be found in the article/Supplementary Material.

## Author Contributions

JP and YX designed this experiment. JP and ZH were responsible for literature review, data collection, analysis, and writing. YX was responsible for modification. All authors contributed to this article and approved the submitted version.

## Conflict of Interest

The authors declare that the research was conducted in the absence of any commercial or financial relationships that could be construed as a potential conflict of interest.

## Footnotes

1 http://cancergenome.nih.gov/

2 https://string-db.org/

3 https://www.rnanut.net/rnam5cfinder/

4 http://www.jci-bioinfo.cn/iRNAm5C-PseDNC

5 http://lin-group.cn/server/iRNA-m5C/service.html

6 http://CIBERSORT.stanford.edu/

7 https://cistrome.shinyapps.io/timer/
