# Supplementary material for: m5C-Related lncRNAs Predict Overall Survival of Patients and Regulate the Tumor Immune Microenvironment in Lung Adenocarcinoma
Source: Front Cell Dev Biol. 2021 Jun 29;9:671821. doi: 10.3389/fcell.2021.671821 (PMC8277384; doi:10.3389/fcell.2021.671821)
Supplement: Supplementary file 1 [file Data_Sheet_1.docx]

Supplementary Material


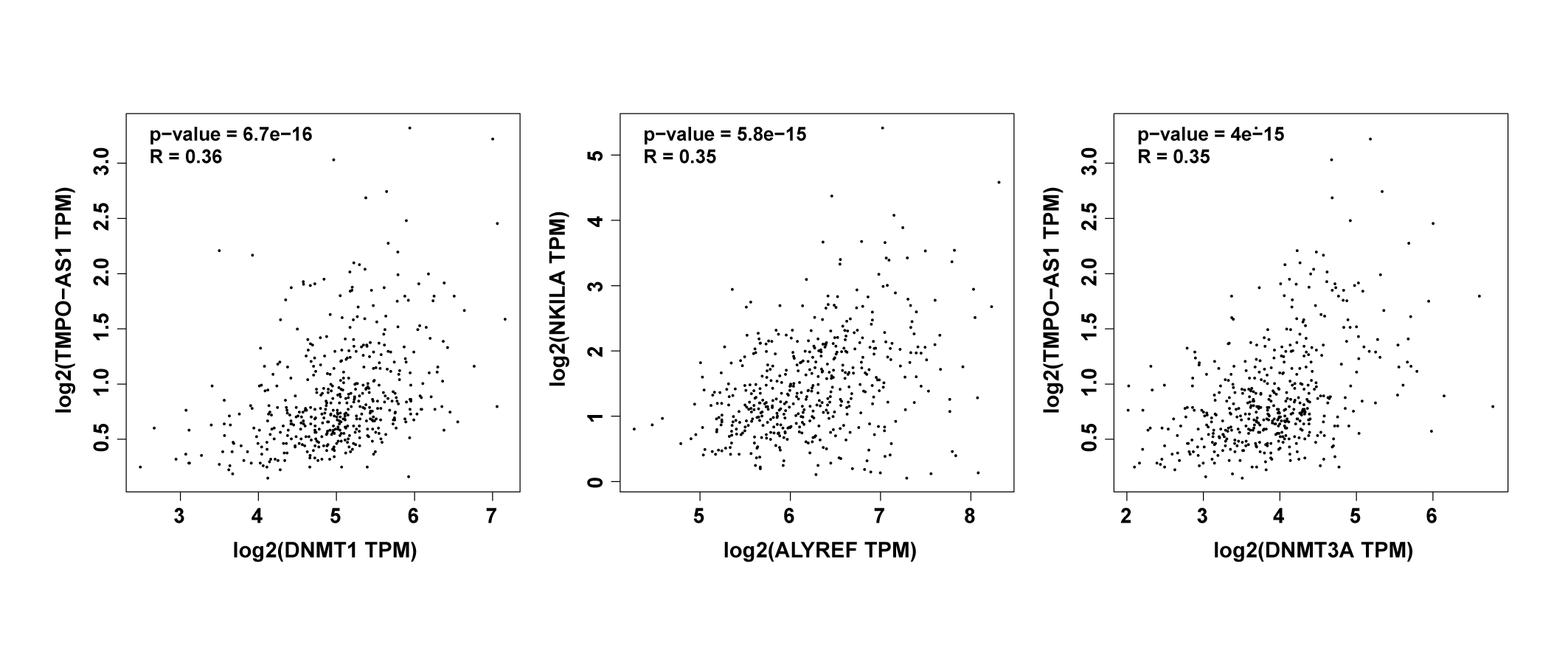


Supplementary Figure 1 Expression Intensity of Related Genes in the Coexpression Network Analyzed Using the Gene Expression Profiling Interactive Analysis(GEPIA) Online Database.


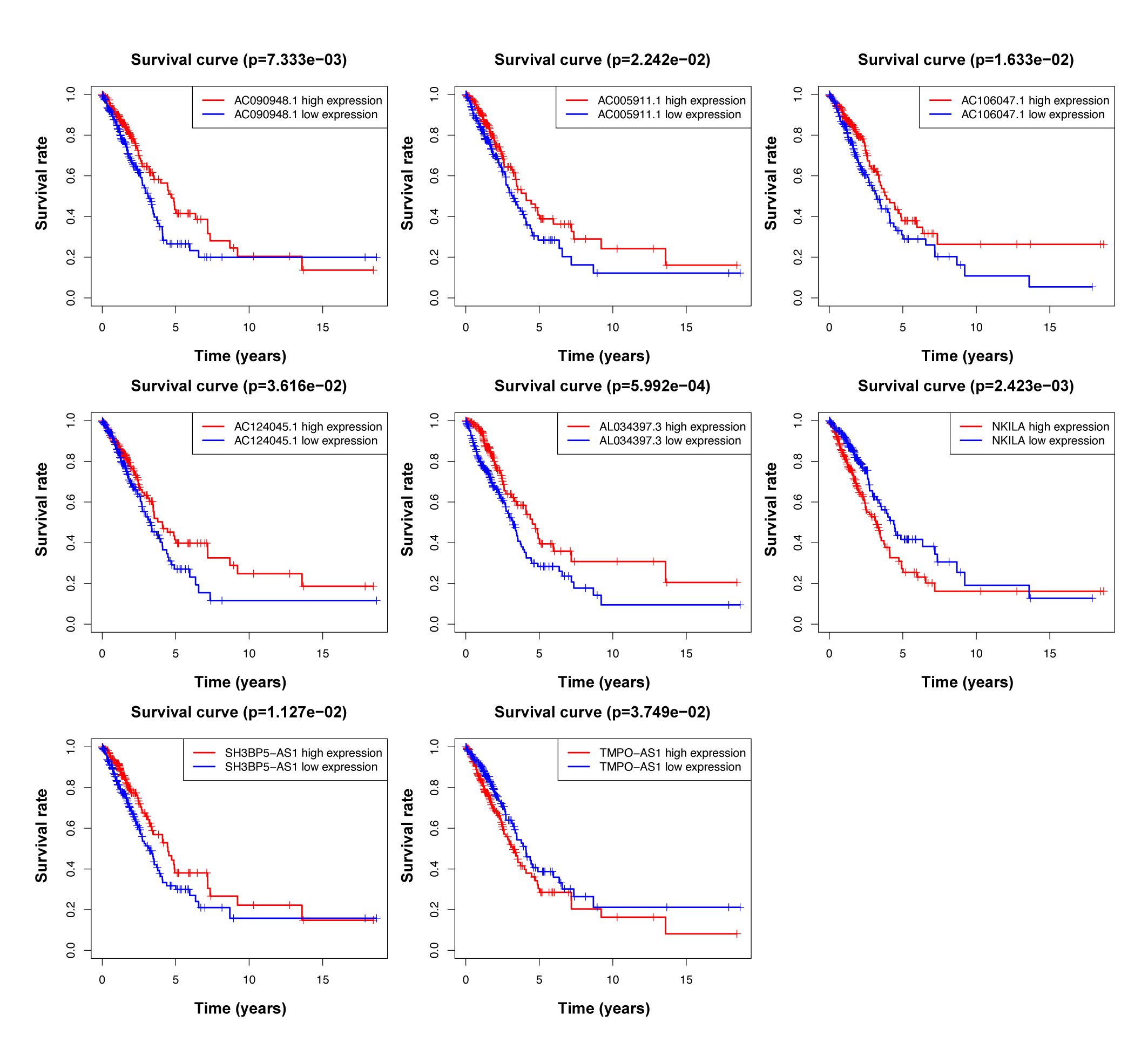


Supplementary Figure 2 Overall Survival (OS) Analysis of the 15 m5C-related lncRNAs in the GEPIA Database Between High- and Low-expression Groups

| Primer | 5' to 3' |
| --- | --- |
| AC124045.1-F | GATAGTCCCAGCATTTACAACCTT |
| AC124045.1-R | AGCCATCTCCAAGACACTGA |
| AC090948.1-F | ACCCTCCACTGTAGACTGATT |
| AC090948.1-R | TTATGTTGGCTGTATTTGCTATGG |
| AL035701.1-F | CCATTGCGAGTGAGTGAGTT |
| AL035701.1-R | AAGCCTGAACAGTCATCTTACAT |
| LINC00578-F | GAGAACCGAAAGGTGCAGAG |
| LINC00578-R | CCCTCACCACACCAAAGAAT |
| AC106047.1-F | TAGAGGGCAGCGAAAGTAATTG |
| AC106047.1-R | GTTCAGGATACAGCACAGAGTC |
| ABALON-F | GCACCAGTGGACTCTGAATC |
| ABALON-R | CCTCTCCCGACCTGTGATAC |
| HLA-DQB1-AS1-F | AGAGTCCAGGGTGTATTGTCAT |
| HLA-DQB1-AS1-R | GCTAGTGGTCGGGAAGAGAT |
| AC005911.1-F | GCTGGGATTATAGGCGTGAA |
| AC005911.1-R | GCCACAGACAGAATACATTAACTC |
| AL513550.1-F | GCTCTTATGTTGACTGCTGTATCC |
| AL513550.1-R | GCCTGTAATCCCTGCTACTTG |
| AL034397.3-F | TTCTCCTGTGTCAATGTCCAATG |
| AL034397.3-R | CTTAGGCAGATGGCTGGTTG |
| SH3BP5-AS1-F | TTGGTGCCTGAACATTAAGAGTAG |
| SH3BP5-AS1-R | CTGTGTCCTTGTCATCCATCTG |
| NKILA-F | AACCAAACCTACCCACAACAC |
| NKILA-R | ACCACTAAGTCAATCCCAGGTG |
| TMPO-AS1-F | AGACGCCGATAAGGGACAG |
| TMPO-AS1-R | AGCCAAGGGTCCTCACA |
| LINC00654-F | AATCTCCCGGCAGTTGGTTC |
| LINC00654-R | TCACCTCCCTGACCACGAAT |
| GAPDH-F | GGTGTGAACCATGAGAAGTATGA |
| GAPDH-R | GAGTCCTTCCACGATACCAAAG |

**Supplementary Table 1 The primer sequences involved in this study.**

| gene | conMean | treatMean | logFC | pValue |
| --- | --- | --- | --- | --- |
| NSUN2 | 8.975555886 | 20.44423591 | 1.187620946 | 1.99E-27 |
| NSUN5 | 4.589599101 | 9.864181506 | 1.103831206 | 3.58E-25 |
| DNMT3B | 0.372217212 | 1.737074689 | 2.222443111 | 1.59E-21 |
| DNMT3A | 2.297156434 | 4.798356124 | 1.062691129 | 1.10E-18 |
| ALYREF | 22.42017192 | 42.74853182 | 0.931077531 | 9.34E-17 |
| DNMT1 | 5.694759963 | 10.14621058 | 0.83323407 | 4.01E-14 |
| NSUN6 | 1.816904549 | 2.688510172 | 0.5653243 | 4.66E-10 |
| TRDMT1 | 0.832071618 | 0.788303722 | -0.077956121 | 3.47E-08 |
| NSUN4 | 2.93226819 | 3.675601885 | 0.325963452 | 1.21E-07 |
| NSUN7 | 1.892180187 | 2.681663344 | 0.503078654 | 2.46E-06 |
| NSUN3 | 1.789613326 | 2.128491526 | 0.250183443 | 0.114115401 |
| YBX1 | 191.9947124 | 191.7752976 | -0.001649679 | 0.163964428 |
| TET2 | 1.797579632 | 2.062452002 | 0.198304862 | 0.724573665 |

**Supplementary Table 2**  Differential Expression of m5C regulators in Lung Adenocarcinoma Tissues and Lung Normal Tissues

| gene | HR | HR.95L | HR.95H | *p*value |
| --- | --- | --- | --- | --- |
| AC024075.2 | 0.850934478 | 0.770514051 | 0.939748581 | 0.001438514 |
| AC124045.1 | 0.662163296 | 0.449237486 | 0.976009895 | 0.037284172 |
| AL022323.1 | 0.81144588 | 0.674289293 | 0.976501367 | 0.026989199 |
| NKILA | 1.102857099 | 1.04917368 | 1.159287357 | 0.000120373 |
| AL035587.1 | 0.691770769 | 0.51660714 | 0.926326333 | 0.01337258 |
| RPARP-AS1 | 0.852402645 | 0.758116059 | 0.958415616 | 0.007582268 |
| AC090948.1 | 0.526726293 | 0.347709759 | 0.797908543 | 0.002483151 |
| AC093673.1 | 1.02667228 | 1.004473425 | 1.049361729 | 0.018266639 |
| KIF26B-AS1 | 0.615507461 | 0.390949047 | 0.969050668 | 0.036106541 |
| AL035701.1 | 0.850867533 | 0.728397743 | 0.993928887 | 0.041673697 |
| LINC00578 | 0.835721339 | 0.748415283 | 0.933212045 | 0.001433495 |
| AL606489.1 | 1.277864183 | 1.130623418 | 1.444280071 | 8.65E-05 |
| AC012615.1 | 0.81869953 | 0.702038588 | 0.954746551 | 0.010760404 |
| AL133445.2 | 0.127143961 | 0.022604881 | 0.715136986 | 0.019261084 |
| TRG-AS1 | 0.711302262 | 0.515912672 | 0.980690987 | 0.037621496 |
| AC004908.3 | 0.7069395 | 0.501112575 | 0.997307755 | 0.048231979 |
| AC067852.3 | 0.58400103 | 0.358216492 | 0.952097993 | 0.031020809 |
| AC106047.1 | 0.689968662 | 0.522141281 | 0.911739356 | 0.00906062 |
| AC083949.1 | 0.583361922 | 0.341605559 | 0.996210757 | 0.048396725 |
| AC123595.1 | 0.486751056 | 0.300181939 | 0.789276635 | 0.003505992 |
| ZNF674-AS1 | 0.830133718 | 0.708485168 | 0.972669606 | 0.021294644 |
| AL050341.2 | 0.919388516 | 0.851414112 | 0.992789796 | 0.03198379 |
| MIR22HG | 0.930145985 | 0.883321399 | 0.979452728 | 0.006000451 |
| AC018809.2 | 0.572296331 | 0.332904649 | 0.983834534 | 0.043495497 |
| ABALON | 1.413852746 | 1.057709698 | 1.889913264 | 0.019341754 |
| AC087752.3 | 0.607045305 | 0.446141883 | 0.825979396 | 0.001489564 |
| AC005856.1 | 0.505146242 | 0.285558039 | 0.893593212 | 0.018948541 |
| ARHGEF26-AS1 | 0.765087131 | 0.601115493 | 0.973786777 | 0.029569556 |
| AC021016.2 | 0.697907061 | 0.502834711 | 0.968656808 | 0.031527137 |
| ATP13A4-AS1 | 0.854640243 | 0.738455283 | 0.989105179 | 0.035126053 |
| AC108134.4 | 0.792591193 | 0.64030458 | 0.981096838 | 0.032738867 |
| VIM-AS1 | 0.815928325 | 0.720402062 | 0.924121498 | 0.001364409 |
| UGDH-AS1 | 0.658535544 | 0.471302564 | 0.920150018 | 0.01438324 |
| AF131215.5 | 0.717074657 | 0.551161429 | 0.932931873 | 0.013248118 |
| AC005070.3 | 0.273979492 | 0.085826064 | 0.874614986 | 0.028802219 |
| AL355075.2 | 0.802632981 | 0.647324866 | 0.995203081 | 0.045092731 |
| MGC32805 | 0.819143216 | 0.699247278 | 0.959597025 | 0.013483396 |
| AC011477.2 | 0.765762894 | 0.661884997 | 0.88594365 | 0.000333057 |
| HLA-DQB1-AS1 | 0.921693122 | 0.882320015 | 0.962823235 | 0.000251433 |
| GMDS-DT | 0.389059202 | 0.159731007 | 0.947637318 | 0.037674904 |
| AC005911.1 | 0.678595675 | 0.474330974 | 0.970824414 | 0.033836448 |
| AL513550.1 | 0.736391445 | 0.560564622 | 0.967368147 | 0.027927248 |
| AC099850.3 | 1.041324755 | 1.016898067 | 1.06633819 | 0.000827018 |
| AL034397.3 | 0.734202131 | 0.590803198 | 0.912406654 | 0.005323413 |
| AC018926.3 | 0.526801008 | 0.309203106 | 0.897530771 | 0.01839189 |
| AC092279.1 | 0.859387622 | 0.757485194 | 0.974998706 | 0.018615838 |
| TMPO-AS1 | 1.308611926 | 1.120036525 | 1.528936901 | 0.000704479 |
| SH3BP5-AS1 | 0.842115533 | 0.741261839 | 0.956691056 | 0.008284983 |
| AC022613.1 | 1.146162864 | 1.055592461 | 1.244504256 | 0.001161678 |
| AC012181.1 | 0.796299348 | 0.640362865 | 0.990208343 | 0.040515382 |
| LINC00654 | 0.754057662 | 0.600383604 | 0.947066099 | 0.015195218 |
| SNHG12 | 0.953150337 | 0.909431198 | 0.998971188 | 0.045184629 |
| AC008764.2 | 0.8528595 | 0.76083024 | 0.956020526 | 0.006295693 |

**Supplementary Table 3** Univariate Cox Regression Analysis Identified m5C-related lncRNAs with Important prognostic Value

|  | **RNAm5Cfinder (score≥0.1)** | **iRNAm5C-PseDNC** | **iRNAm5C** |
| --- | --- | --- | --- |
| AC124045.1 | 6 | 0 | 240 |
| AC090948.1 | 4 | 0 | 61 |
| AL035701.1 | 8 | 4 | 248 |
| LINC00578 | 7 | 0 | 141 |
| AC106047.1 | 2 | 0 | 46 |
| ABALON | 43 | 14 | 311 |
| HLA-DQB1-AS1 | 7 | 0 | 73 |
| AC005911.1 | 7 | 0 | 52 |
| AL513550.1 | 2 | 15 | 203 |
| AL034397.3 | 7 | 0 | 219 |
| SH3BP5-AS1 | 34 | 11 | 692 |
| NKILA | 41 | 2 | 319 |
| TMPO-AS1 | 39 | 17 | 389 |
| LINC00654 | 11 | 0 | 300 |

RNAm5Cfinder , iRNAm5C-PseDNC , iRNAm5C online databases were mainly used to predict m5C modification sites in coding RNA with some deviation in predicting in non-coding RNA.

**Supplementary Table 4** Number of m5C Modification Sites on Prognostic-related lncRNAs
